# Characteristics and management of congenital esophageal stenosis: findings from a multicenter study

**DOI:** 10.1186/1750-1172-8-186

**Published:** 2013-12-01

**Authors:** Laurent Michaud, Frédéric Coutenier, Guillaume Podevin, Arnaud Bonnard, François Becmeur, Naziha Khen-Dunlop, Frédéric Auber, Aude Maurel, Thomas Gelas, Martine Dassonville, Corinne Borderon, Alain Dabadie, Dominique Weil, Christian Piolat, Anne Breton, Djamal Djeddi, Alain Morali, Florence Bastiani, Thierry Lamireau, Frédéric Gottrand

**Affiliations:** 1 Reference Centre for Congenital and Malformative Esophageal Diseases, Lille, France; 2 Pediatric Surgery Department, Nantes, France; 3 Pediatric Surgery Department, Paris Robert Debré, France; 4 Pediatric Surgery Department, Strasbourg, France; 5 Pediatric Surgery Department, Paris Necker, France; 6 Pediatric Surgery Department, Paris Trousseau, France; 7 Pediatric Surgery Department, Saint Pierre de la Réunion, France; 8 Pediatric Surgery Department, Lyon, France; 9 Pediatric Surgery Department, Bruxelles, Belgium; 10 Pediatric Department, Clermont Ferrand, France; 11 Pediatric Department, Rennes, France; 12 Pediatric Surgery Department, Angers, France; 13 Pediatric Surgery Department, Grenoble, France; 14 Pediatric Department, Toulouse, France; 15 Pediatric Department, Amiens, France; 16 Pediatric Surgery Department, Nancy, France; 17 Pediatric Surgery Department, Nice, France; 18 Pediatric Department, Bordeaux, France

**Keywords:** Congenital esophageal stenosis, Esophageal atresia, Children, Endoscopic dilatation

## Abstract

**Background:**

Congenital esophageal stenosis (CES) is a rare condition frequently associated with esophageal atresia (EA). There are limited data from small series about the presentation, treatment, and outcomes of CES.

**Methods:**

Medical records of all patients with CES included in the French Network on Esophageal Malformations and Congenital Diseases were reviewed retrospectively with regard to diagnosis, treatment, and outcome.

**Results:**

Over 18 years, 61 patients (30 boys) had CES, and 29 (47%) of these patients also had EA. The mean age at diagnosis was 24 months (1 day to 14 years) and was younger in patients with CES and EA than in those with isolated CES (7 vs. 126 months, p < 0.05). Twenty-one of the 61 patients with CES had no clinical symptoms: in three patients, the findings were incidental, and in 18 of the 29 patients with associated EA, CES was diagnosed at the time of surgical repair of EA or during a postoperative systematic esophageal barium study. In the 40 other patients, at diagnosis, 50% presented with dysphasia, 40% with vomiting, 50% with food impaction, and 42% with respiratory symptoms. Diagnosis of CES was confirmed by esophageal barium study (56/61) and/or esophageal endoscopy (50/61). Sixteen patients had tracheobronchial remnants (TBR), 40 had fibromuscular stenosis (FMS), and five had membrane stenosis (MS). Thirty-four patients (56%) were treated by dilation only (13/34 remained asymptomatic at follow-up); 15 patients were treated by dilation but required later surgery because of failure (4/15 remained asymptomatic at follow-up); and nine patients had a primary surgical intervention (4/9 were asymptomatic at follow-up). Dilation was complicated by esophageal perforation in two patients (3.4%). At follow-up, dysphagia remained in 36% (21/58) of patients, but the incidence did not differ between the EA and the isolated CS groups (10/29 vs. 7/32, p = 0.27).

**Conclusions:**

CS diagnosis can be delayed when associated with EA. Dilation may be effective for treating patients with FMS and MS, but surgical repair is often required for those with TBR. Our results show clearly that, regardless of the therapeutic option, dysphagia occurs frequently, and patients with CES should be followed over the long term.

## Background

Congenital esophageal stenosis (CES) is a very rare clinical condition found in 1 per 25,000 to 50,000 live births, although the true incidence remains unknown. CES is characterized by an intrinsic circumferential narrowing of the esophageal lumen that it is present at birth, although not necessarily symptomatic in the neonatal period [1]. Its etiology remains unknown, but an embryologic origin has been suggested. There are three histological types of CES: ectopic tracheobronchial remnants in the esophageal wall (TBR), segmental fibromuscular hypertrophy of the muscle and submucosal layers (FMS), and a membranous diaphragm or stenosis (MS) [2]. CES is frequently associated with esophageal atresia (EA) [3,4]. The definitive preoperative diagnosis is often difficult, and there are few data about treatment and outcomes from small series. The aim of this study was to assess the circumstances of diagnosis, management, and outcomes of CES in a large multicenter cohort.

## Methods

All 38 participating centers of the French Network on Esophageal Malformations and Congenital Disease were asked to search in their databases for patients treated for CES in their institution during the past 18 years. Sixty-one patients with CES were identified. The data were obtained retrospectively from the patients’ clinical, radiological, endoscopic, and operative records. We analyzed clinical characteristics including sex, age at diagnosis, clinical symptoms at presentation, and associated malformations. Ladd’s classification of EA was used when appropriate. The management and outcomes of CES were also reviewed. We collected histological information when available.

### Statistical analysis

The data were compared between the patients with isolated CES and those with CES associated with EA using the χ^2^ test or Wilcoxon test. A p value < 0.05 was considered significant.

## Results

Sixty-one patients were diagnosed with CES during the study period. The female-to-male ratio was 1 (30 boys, 31 girls). At the time of diagnosis, the patients’ ages ranged from 1 day to 14 years (mean age at diagnosis: 2 years); seven (11%) with CES were diagnosed after the age of 5 years. In 29/61 patients (48%), CES was associated with type III EA (EA associated with a distal tracheoesophageal fistula).

### Clinical symptoms at presentation

Patients with CES associated with EA were younger at the time of diagnosis than were patients with isolated CES (7 *vs* 126 months, p < 0.05). Twenty-one patients with CES (34%) did not present with any symptom at the time of diagnosis: 18 (of 29 with associated EA) were diagnosed at the time of surgical repair of EA or postoperatively at the time of control esophageal opacification, and in three patients the findings were incidental. For one of the patients whose finding was incidental, CES was diagnosed at birth in the maternity ward when a nasogastric tube was passed for desobstruction; for the other two patients with associated EA whose findings were incidental, CES was diagnosed endoscopically during follow-up of EA.

Forty of the 61 children with CES (66%) presented with symptoms at a mean age of 39 months (range 1 to 166 months). Dysphagia (50%), food impaction (50%), and repeated vomiting (40%) were the most frequent symptoms. For six patients, CES was revealed during examination of an esophageal retained foreign body. In addition to gastrointestinal symptoms, respiratory signs (respiratory failure, dyspnea) were observed in 27% of patients. At diagnosis, 35% of the patients were classified as malnourished (Z-score weight for height < -2 SD). Repeated vomiting, food impaction, and impaired growth were more frequently observed as revealing symptoms in the group with isolated CES than in patients with CES associated with EA (all p < 0.05).

### Diagnosis of CES

**Figure 1:**
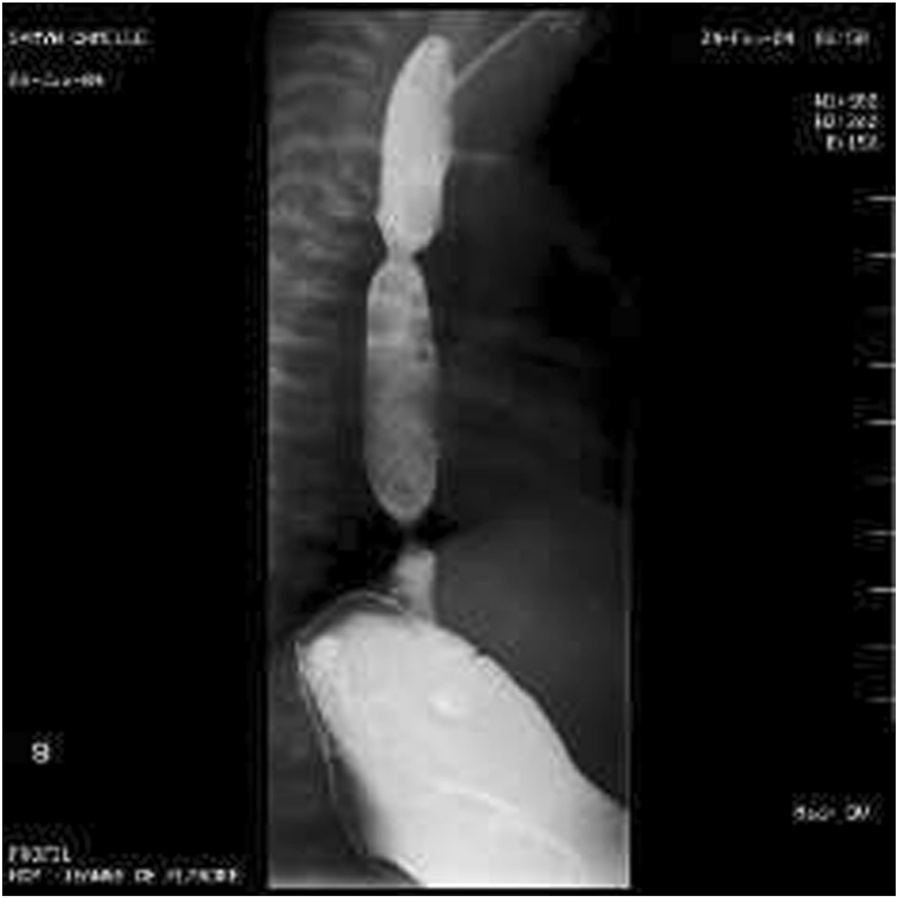
**CES in the lower esophagus in a child with EA.** Esophageal barium study.

**Figure 2:**
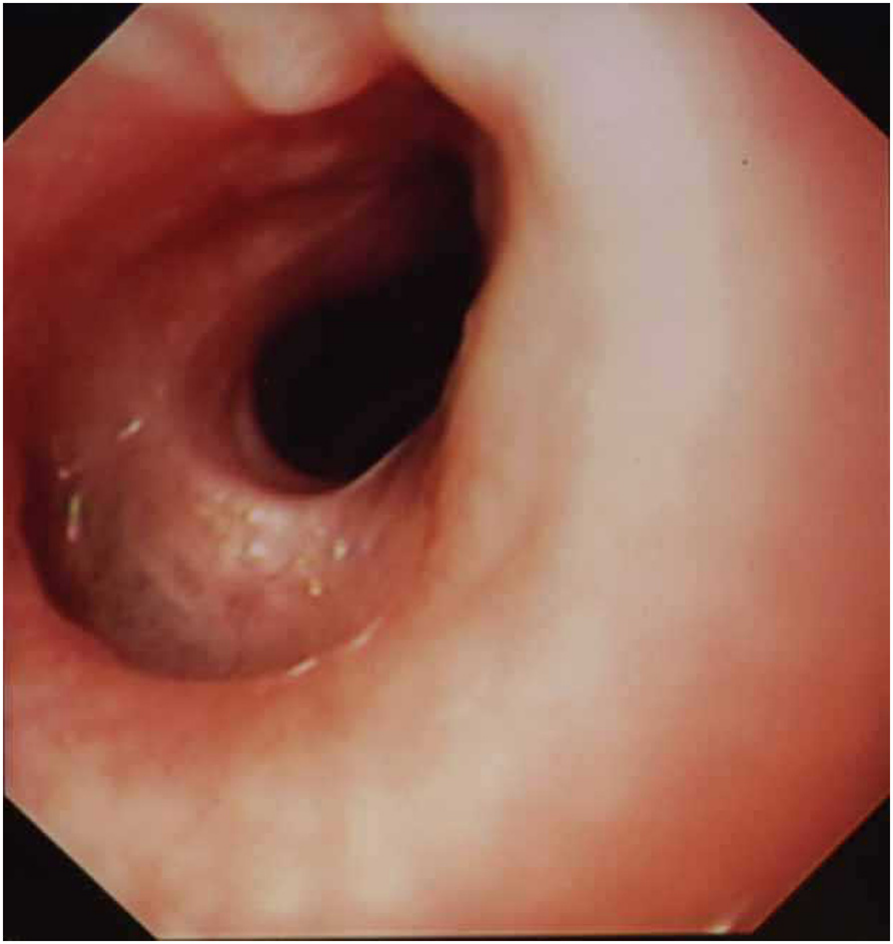
Endoscopic aspect of a CES.

An esophageal barium study identified esophageal stenosis in 56/61 patients (91%), (Figure 1). Endoscopy performed in 50/61 patients (82%) confirmed the diagnosis in all patients with stenosis associated with normal aspects of the mucosa (Figure 2). Endoscopic ultrasonography was performed in only one patient. The stenosis was located at the level of the gastroesophageal junction in three patients, 1 to 2 cm above the gastroesophageal junction in 40 patients, in the median part of the esophagus in 12, and in the upper part of the esophagus in six patients (Table 1). Computed tomography scanning was performed in eight patients and confirmed all CES diagnoses but did not show any signs of TBR in the esophageal wall.

**Table 1:** Location of CES

	**Upper esophagus**	**Middle esophagus**	**Lower esophagus**	**Cardia**
Isolated CES (n = 32)	6 (19%)	6 (19%)	18 (56%)	2 (6%)
CES associated with EA (n = 29)	0 (%)	6	22	1
All patients (n = 61)	6 (10%)	12 (20%)	40 (65%)	3 (5%)

Pathology examination of the stenosis was available in 18 patients at the time of operation. TBR comprising either mature or immature cartilage was found in 12 patients. Fibromuscular thickening and circumferential proliferation of the smooth muscle fibers were found in the other six patients. In absence of histological confirmation of TBR stenosis and in absence of endoscopic aspect of membrane stenosis (MS), diagnostic of FMS was usually retained, probably leading to an overestimation of the number of FMS.

Using endoscopic and histological data when available, the type of stenosis was classified as TBR (n = 16), FMS (n = 40), or MS (n = 5). No patient presented with multiples stenoses. MS was not found in any patient with EA.

### Treatment and outcomes

**Figure 3:**
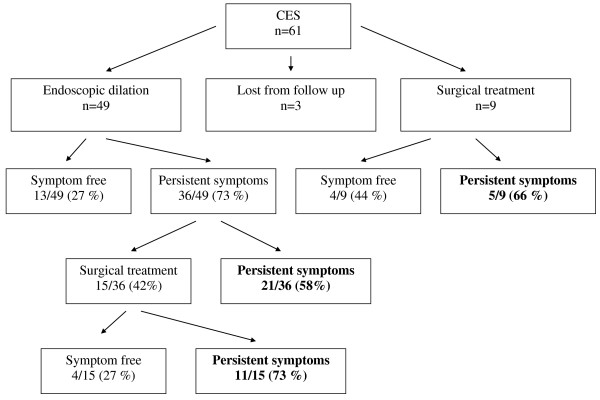
Treatment and outcomes.

Esophageal dilation was the first-line treatment of 49/58 patients (84%), (Figure 3). Data concerning treatment were missing for three patients lost to follow-up after the initial diagnosis. Savary bougienage (39 sessions) and/or balloon dilation (103 sessions) were performed under general anesthesia. Since no technique have been shown to be superior to the other, the choice of dilation technique (bouginage or hydrostatic dilation) depended on personal experience or preference. The median number of dilations per patient was 2.5 (range: 1 to 11). Sixteen patients had Savary bouginage dilation (3 to 5 dilations per patients), 35 were treated with balloon dilation (2 to 11 dilations per patients). Two patients had successively Savary and balloons dilation. Esophageal perforation occurred in two patients (3.4%). One perforation occurred after Savary dilation, the other after balloon dilation. The first patient who presented perforation after Savary dilation was not operated first. Conservative treatment (IV antibiotic and parenteral nutrition) was effective but this patient needs finally a surgical resection. The other patient who presented esophageal perforation after balloon dilatation (8 sessions) had surgical resection and coloplasty.

Thirteen of the 49 patients (27%) became free from symptoms after endoscopic dilation, whereas 36/49 (73%) had persistent symptoms (moderate to severe dysphagia); 15 (30%) required additional surgical treatment (Figure 2).

Nine patients (16%) received first-line surgical treatment; four became free from symptoms, but the other five had persistent dysphagia at the follow-up 1 to 18 years later. Fifteen patients were operated because of persistent symptoms after dilatation. Four patients were symptom free, while 11 of them had persistent symptoms after surgery. In total, 24 patients underwent surgical treatment (Figure 3). Surgical treatment comprised resection of the stenotic segment and end-to-end esophageal anastomosis. Five out of 9 who underwent first line surgery and 11/15 operated because of endoscopic dilation failure remained symptomatic after surgery. At total 16/24 (66%) of surgical patients remained symptomatic after surgery. In those children presenting persistent dysphagia we cannot definitively separate persistent esophageal stricture from esophageal dysmotility.

At follow-up (median: 33 months; range: 1 months to 20 years), dysphagia persisted in 64% (37/58) of patients regardless of the treatment. The incidence did not differ between the group with CES and EA and the isolated CES group (10/29 *vs* 7/32; p = 0.27) (Table 1). All 15 patients with TBR for whom follow-up was available underwent operative repair; 10 were treated by esophageal dilatation before surgery.

## Discussion

To our knowledge, our study is the largest reported and thus provides, for the first time, a description of the circumstances of diagnosis and the outcomes of this very rare malformation. Multiple CES lesions, the rarest form of this anomaly, were not observed in our series, and only a few cases have been reported [5,6]. CES without associated EA is diagnosed rarely in the neonatal period because symptoms usually start after the introduction of solid foods, and diagnosis can be delayed into the second decade of life, as found in our series. The classical revealing circumstance is food impaction, but other signs may precede this, such as failure to thrive, aspiration pneumonia, and dysphagia, as we observed in our series. Of interest, we found that the diagnosis of isolated CES can be made during the initial examination in the maternity ward when a gastric tube test is used to rule out EA, as we observed in one patient.

CES associated with EA is diagnosed in only 62% (18/29) of patients at the time of the initial esophageal surgical repair, meaning that even in patients with symptoms of EA, the diagnosis of CES can be delayed. In addition to the numerous causes of feeding problems in EA (e.g., motility disorders, esophagitis, and anastomotic stenosis), CES should be considered when symptoms of dysphagia and food impaction persist in children after EA repair. A postoperative esophageal barium study is helpful for revealing this lesion [7].

Conservative treatment (dilation with a Savary bougie or balloon) is used as the first-line treatment in most cases (49/58), but only 27% (13/49) of our patients became asymptomatic after endoscopic dilation. Persistent dysphagia may indicate failure of dilation, but it can also be related to esophageal dysmotility observed in patients with CES associated EA or with isolated CES [8].

Despite the lack of large published series, some authors have suggested that the therapeutic approach for CES should depend on the etiology and that dilation is ineffective in patients with TBR, and that therefore these patients should undergo operative repair [6]. In our series, all patients with TBR had first- or second-line surgical treatment that confirmed the presence of the TBR form of CES, which is resistant to dilation. Of interest, all perforations occurred in children with TBR, reinforcing the need for first-line surgery in patients with this form of CES. However, because we did not have a histological analysis of the lesions for the patients who responded to dilation, we cannot formally exclude the possibility that TBR can respond to esophageal dilation. Computed tomography scanning (CTS) was indeed performed in eight patients and confirmed all CES diagnoses but did not show any signs of tracheobronchial remnants in the esophageal wall, and so did not allow to diagnose TBR-CES. CTS is not a good method to differentiate TBR-CES and fibromuscular stenosis (FMS) CES. Miniprobe endoscopic esophageal ultrasound is a promising method to discriminate FMS from TBR [9], but this method was not used in this series. The definitive preoperative diagnosis of CES is often difficult to make. The definition and classification of CES proposed by Nihoul-Fékété was used in our study. This classification delineates 3 forms of CES: tracheobronchial remnants, fibromusclar stenosis and membranous diaphragm in the wall of the esophagus. In absence of histological confirmation of TBR stenosis and in absence of endoscopic aspect of membrane stenosis (MS), diagnostic of FMS was retained, probably leading to an overestimation of the number of FMS. We cannot exclude TBR in the group of patients in which the dilations were effective.

Since there is at present time no consensus on treatment of CS, the type of initial treatment (surgical or endoscopic) varied according to the teams, and also depended if the patient was in a surgical or a gastroenterological unit.

In patients with FMS or suspected FMS, balloon dilation or bougienage may be the treatment of choice because dilation was effective in 38% of our patients without the need for second-line surgery and without causing any complications. Some authors have reported the addition of endoscopic incision or partial resection of the diaphragm to esophageal dilatation [10,11], but we found these to be of no use in our study.

Finally, an important finding of our study is that surgery should not been considered a definite curative option because 66% (16/24) of our patients remained symptomatic after surgery (Figure 3). Our results show clearly that, regardless of the therapeutic option, dysphagia often remains and suggest that patients with CES should be followed over the long term.

## Abbreviations

CES: Congenital esophageal stenosis; EA: Esophageal atresia; TBR: Tracheobronchial remnants; FMS: Fibromuscular stenosis; MS: Membrane stenosis; CTS: Computed tomography scanning.

## Competing interests

The authors declare that they have no competing interests.

## Authors’ contributions

FC coordinated the study and drafted the manuscript; LM and FG participated in the design of the study, sample analysis, data analysis, and drafting of the manuscript; FC, GP, AB, FB, NK, FA, AM, TG, MD, CB, AD, DW, CP, AB, DD, AM, FB, and TL recruited the patients, contributed to collection of data, and reviewed the draft of the manuscript. All authors read and approved the final manuscript.
